# Red Flag/Blue Flag visualization of a common CNN for text classification

**DOI:** 10.1093/jamiaopen/ooac112

**Published:** 2023-01-16

**Authors:** John Del Gaizo, Jihad S Obeid, Kenneth R Catchpole, Alexander V Alekseyenko

**Affiliations:** Biomedical Informatics Center, Medical University of South Carolina, Charleston, South Carolina, USA; Biomedical Informatics Center, Medical University of South Carolina, Charleston, South Carolina, USA; Department of Anesthesia and Perioperative Medicine, Medical University of South Carolina, Charleston, South Carolina, USA; Biomedical Informatics Center, Medical University of South Carolina, Charleston, South Carolina, USA; Department of Public Health Sciences, Medical University of South Carolina, Charleston, South Carolina, USA; Department of Oral Health Sciences, Medical University of South Carolina, Charleston, South Carolina, USA; Department of Healthcare Leadership and Management, Medical University of South Carolina, Charleston, South Carolina, USA

**Keywords:** explainable AI, X-AI, CNN, NLP, text classification, clinical NLP

## Abstract

A shallow convolutional neural network (CNN), TextCNN, has become nearly ubiquitous for classification among clinical and medical text. This research presents a novel eXplainable-AI (X-AI) software, Red Flag/Blue Flag (RFBF), designed for *binary* classification with TextCNN. RFBF visualizes each convolutional filter’s discriminative capability. This is a more informative approach than direct assessment of logit contribution, features that overfit to train set nuances on smaller datasets may indiscriminately activate large logits on validation samples from both classes. RFBF enables model diagnosis, term feature verification, and overfit prevention. We present 3 use cases of (1) filter consistency assessment; (2) predictive performance improvement; and (3) estimation of information leakage between train and holdout sets. The use cases derive from experiments on TextCNN for binary prediction of surgical misadventure outcomes from physician-authored operative notes. Due to TextCNN’s prevalence, this X-AI can benefit clinical text research, and hence improve patient outcomes.

## Introduction

Red Flag/Blue Flag (RFBF) is eXplainable-AI (X-AI) software to visualize a convolutional neural network (CNN) architecture, TextCNN,^1^ that is the “standard baseline for new text classification architectures.”^2^ RFBF is written in Python 3.10 and supports a PyTorch^3^ implementation of TextCNN.^1^

Despite model simplicity, recent researches indicate strong classification performance^2^^,^^4–8^; with superior results to most deep learning models,^2^^,^^8^ and similar results to memory-intensive transformers.^2^^,^^8^^,^^9^ Lu et al^8^ show TextCNN^1^ to outperform 5 other common network models—including BERT^9^—for 16 binary classification tasks from discharge notes, with one-tenth the training time of BERT.

This research is influenced by 3 X-AI techiques^10–12^ developed for TextCNN.^1^

1. Both this research and Jacovi et al^11^ present a *model interpretability* through display of a filter activation grid—a textual analogue to the Zeiler–Fergus (ZF)^13^ display. However, RFBF displays differences in filter logit output between outcomes. A comparison of these interclass logit differences across datasets provides additional insights into model behavior.
2. Cheng et al^12^ developed a *sample interpretability* technique to calculate each token’s contribution to a prediction. The derivation served as a basis for this research.
3. Zhao et al^10^ present phrase-level SHAP^14^ (Shapley Additive exPlanations) features for both model and sample interpretability. The authors rank phrases with methods that account for frequency and SHAP magnitude. Our X-AI is complimentary: RFBF focuses on model filters instead of phrases, and the interclass difference in feature contribution to logit outputs instead of SHAP values.

In summary, a novel aspect to RFBF is that filter concepts are presented through interclass logit differences, as opposed to solely logit magnitudes.

## METHODS

### TextCNN architecture

**Figure 1.**
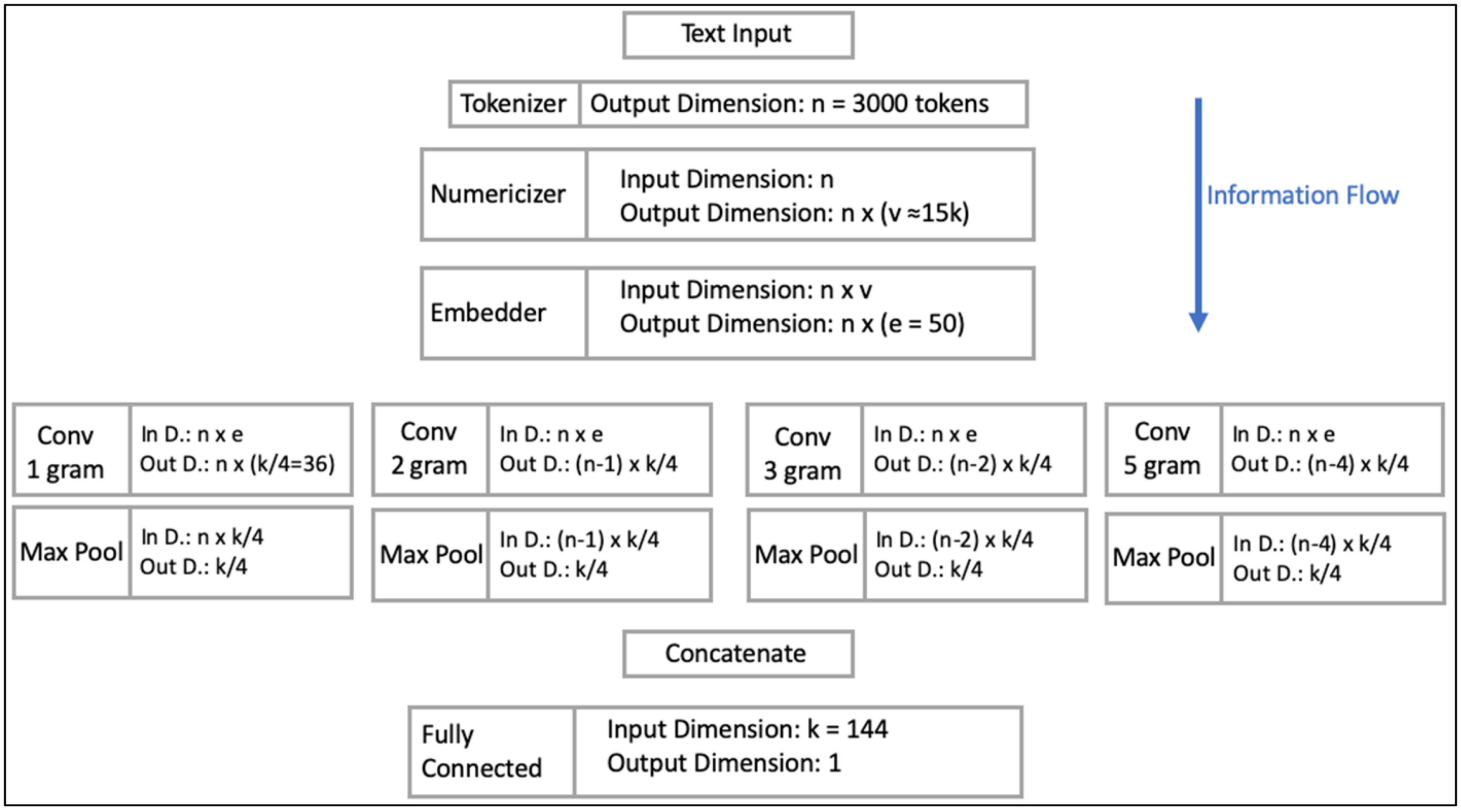
Modified TextCNN. The sole difference with the original TextCNN is that the FC layer outputs 1 dimension instead of 2.

Previous researches explain the TextCNN architecture^1^ (Figure 1) in detail.^10–12^ We highlight the following layers:

1. *Convolution layer:* convolution with *k* filters, $R^{n\times e}\to R^{n\times k}$, which are split across different ngram sub-layers. The subsequent max pool *scans* the text input for the ngram that most closely resembles the filter to output 1 activation per filter, $R^{n\times k}\to R^{k}$. A moniker for TextCNN^1^ could be “scan-CNN.”
2. *Fully connected (FC) layer*: outputs 2 logits for binary prediction, $R^{k}\to R^{2}$.

We utilize 144 filters: 36 length-1 filters (unigram), 36 length-2 filters (bigram), 36 length-3 filters (trigram), and 36 length-5 filters (5-gram).

### TextCNN architecture modifications

For binary classification, the FC layer of TextCNN^1^ can be modified to output a single logit, $R^{k}\to R$, instead of 2 (Figure 1).

The modified architecture has equivalent capacity: a function that is modeled as 2 logits passed to a softmax can be represented with a single logit and its negation passed to a softmax; where the single logit is the halfway distance between the 2 original logits.

$$\begin{array}{l} P\left(1\right)=SM\left(\mathrm{logi}t_{1};\mathrm{logi}t_{0}\right)=\frac{e^{\mathrm{logi}t_{1}}}{e^{\mathrm{logi}t_{0}}+e^{\mathrm{logi}t_{1}}}\\ l\mathrm{ogi}t_{\mu }=\frac{\mathrm{logi}t_{1}+\mathrm{logi}t_{0}}{2} \end{array}$$

$$\mathrm{logi}t_{\delta }=\frac{\mathrm{logi}t_{1}-\mathrm{logi}t_{0}}{2}=\mathrm{logi}t_{1}-\mathrm{logi}t_{\mu }=\mathrm{logi}t_{\mu }-\mathrm{logi}t_{0}$$

$$\begin{array}{l} P\left(1\right)=\frac{e^{\mathrm{logi}t_{1}-\mathrm{logi}t_{\mu }}}{e^{\mathrm{logi}t_{0}-\mathrm{logi}t_{\mu }}+e^{\mathrm{logi}t_{1}-\mathrm{logi}t_{\mu }}}\\ =\frac{e^{\mathrm{logi}t_{\delta }}}{e^{-\mathrm{logi}t_{\delta }}+e^{\mathrm{logi}t_{\delta }}}\\ =SM\left(\mathrm{logi}t_{\delta };-\mathrm{logi}t_{\delta }\right) \end{array}$$

The modification results in a bijective relationship between the K fully connected weights and K convolutional filters, which facilitates visualization since each filter contributes to one class. In contrast, each original TextCNN^1^ filter contributes to both classes. However, the 2-d FC weights of post-trained TextCNN^1^ models can be converted to 1-d weights as halfway distances, with the logit output and it’s negation passed to softmax.

Each filter outputs a logit, $l_{k}$. The filter logits are summed to output a sample logit.

$$\mathrm{logit}=\sum^{K}l_{k}\;+\;\mathrm{bias}$$

The CNN is therefore an ensemble summation of *K* classifiers, where each filter is a classifier. The *bias* does not affect interclass difference metrics such as Area Under Curve (AUC).

Only the ngram section at location *j* that passes the max pool for filter *k*, $x_{j:j+n}^{k}$, contributes to the filter’s logit, $l_{k}$. This activation is calculated via dot product, $x_{j:j+n}^{k}\cdot w_{k}$, which is scaled by $FC_{k}$.

$$\begin{array}{l} l_{k}=x_{j:j+n}^{k}\cdot w_{k}\times FC_{k}\\ l\mathrm{et}\;w_{k}=u_{k}\times ||w_{k}||\\ \begin{array}{l} =x_{j:j+n}^{k}\cdot u_{k}\times ||w_{k}||\times FC_{k}\\ =x_{j:j+n}^{k}\cdot u_{k}\times ||w_{k}||\;\times |FC_{k}|\;\times \mathrm{sign}\left(FC_{k}\right)\\ \begin{array}{l} l\mathrm{et}\;Imp_{k}=||w_{k}||\times |FC_{k}|\\ =x_{j:j+n}^{k}\cdot u_{k}\times Imp_{k}\times \mathrm{sign}\left(FC_{k}\right)\\ \begin{array}{l} l\mathrm{et}\;x_{j:j+n}^{k}=e_{j:j+n}^{k}\times ||x_{j:j+n}^{k}||\\ =e_{j:j+n}^{k}\cdot u_{k}\times ||x_{j:j+n}^{k}||\times Imp_{k}\times \mathrm{sign}\left(FC_{k}\right)\\ =\text{cos}\;\left(\theta_{e->u}\right)\times ||x_{j:j+n}^{k}||\times Imp_{k}\times \mathrm{sign}\left(FC_{k}\right) \end{array} \end{array} \end{array} \end{array}$$

The 4 multiplicative components of $l_{k}$:

- *Cosine similarity*, $\theta_{e\to u}$ is the angle between the ngram embedding, $e_{j:j+n}^{k}$, and convolution filter, $u_{k}$.
- *Embedding magnitude*, $\left||x_{j:j+n}^{k}|\right|$
- *Sample-independent filter importance*, $Imp_{k}=||w_{k}||\times |FC_{k}|$
- *Class membership*: $\mathrm{sign}\left(FC_{k}\right)$

To reduce clutter, the convolution kernel biases are not notated. These biases do not affect interclass performance and can theoretically be summed into the *FC* bias after multiplication with $FC_{k}$. However, they are retained to facilitate training.

The max pool excludes negative activations in this data, which would flip the classification of $FC_{k}$. A pre-FC ReLU can exclude them in other datasets.

### Logit deltas

Discriminative capability depends on the distributional *difference* (delta) in logit outputs between outcome classes. For small or noisy datasets, direct interpretation of filter logits on unseen data—without consideration of discriminative capability—may emphasize overfit features associated with large logits from both classes.

To reflect discriminative capability, RFBF displays each filter’s (1) AUC; and (2) difference in the median/mean logit value between patient and control sets:

$$\Delta_{k,\mathrm{med}}=\mathrm{med}\left({\{l}_{k}^{i}\mathrm{for}i\in y_{i}=1\}\right)-\mathrm{med}\left(\left\{l_{k}^{i}\mathrm{for}i\in y_{i}=0\right\}\right)$$

$$\Delta_{k,\mathrm{mean}}=\mathrm{mean}\left(\left\{l_{k}^{i}\mathrm{for}i\in y_{i}=1\right\}\right)-\mathrm{mean}\left(\left\{l_{k}^{i}\mathrm{for}i\in y_{i}=0\right\}\right)$$

### Visualization

**Figure 2.**
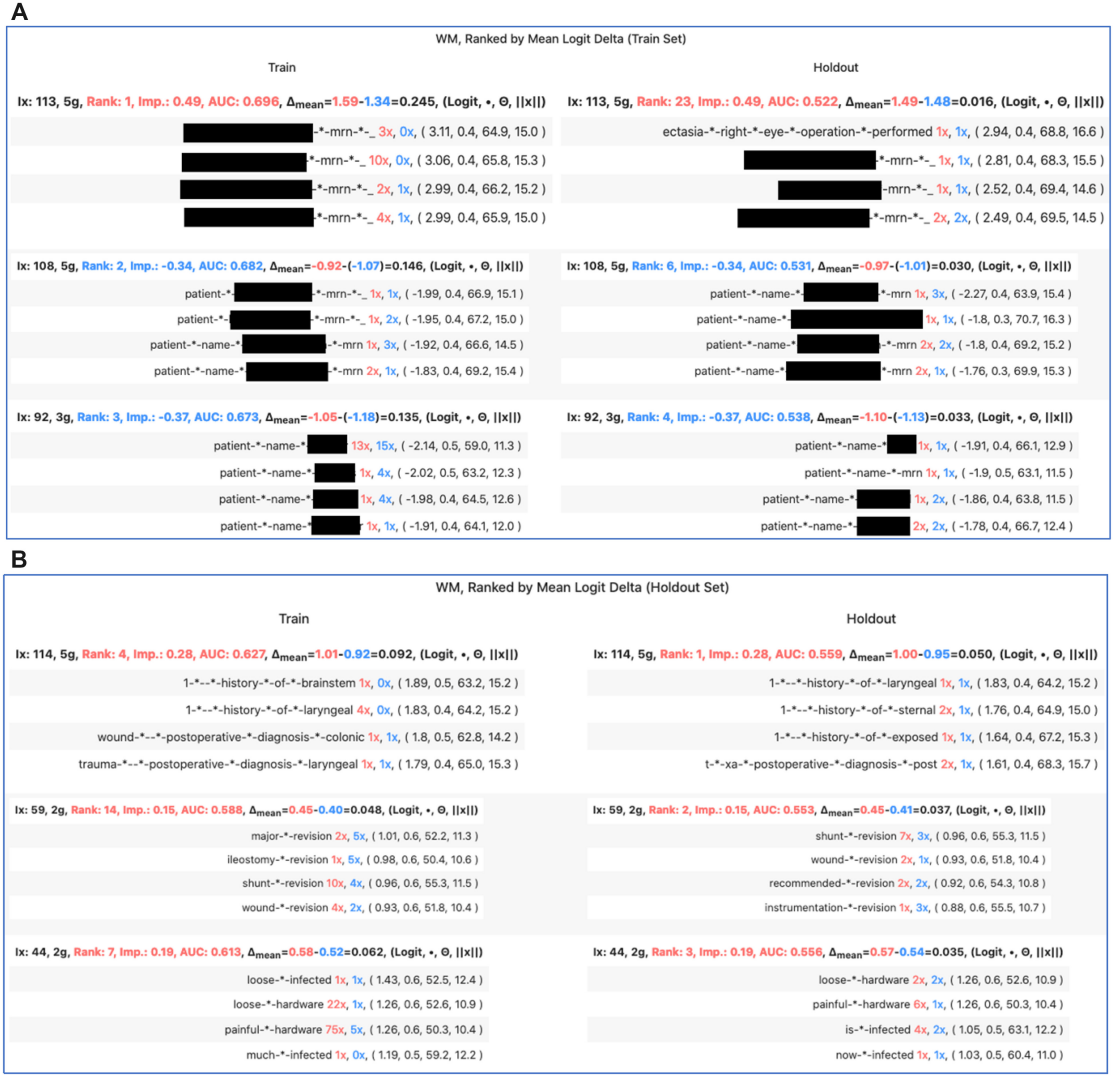
(A) The top 3 filters ranked on mean logit delta (train set) for classification of without mention. (B) Same as Figure 2A but ranked on mean logit delta (holdout set).

RFBF outputs an HTML table. Each table row corresponds to a filter, and the 2 columns to data subsets, i.e. train and validate. Each table cell portrays the top 4 activated ngrams for the associated filter (row) and dataset (column), and the following information for each ngram (Figure 2):

- The number of ngram instances in the dataset as a patient (red), and control (blue)
- Logit
- Cosine similarity between embedding and filter
- embedding magnitude

Each cell heading portrays ngram-independent information:

- The filter’s index, *k*, in the classification layer.
- The filter rank according to sort options
- AUC
- Logit delta and calculation inputs
  - Patient logit: The median or mean (user specified) logit in the patient set, in red.
  - Control logit: analogous to patient logit, in blue.
  - Delta: patient logit minus control logit
-
$Imp_{k}=||w_{k}||\times |FC_{k}|$ , red for patient ($\mathrm{sign}\left(FC_{k}\right)=1$), otherwise blue

RFBF provides options to rank the filters via discriminative performance (mean/median logit delta, AUC) on the first or second dataset; or by filter importance, $Imp_{k}$.

The RFBF user-interface follows a 2-step process. (1) Invoke the function *calc_zf_dict* on the CNN model and the train, validate, and holdout datasets to obtain 3 ZF^13^ dictionaries. The keys are filter indices in the classification layer. For example: our classification layer has 144 weights, hence the indices range from 0 to 143. (2) Input any 2 of the 3 ZF objects to the *make_zfs_table* function.

The 2-step design enables the user to filter, graph, and perform exploratory analyses of the ZF dictionaries.

### Data

We experiment with prediction of surgical misadventure from physician-authored operative notes obtained across diverse surgical types at the Medical University of South Carolina between May 2015 and July 2019. The control set is composed of 6800 notes from 5135 controls. The patient dataset is composed of 2 subsets: (1a) *WM* outcome—13 254 notes from 7628 patients who had an ICD-10 code for “adverse event *without mention* at time of operation” (Y83–Y84.9); and (1b) *D&I* outcome—840 notes from 537 patients who had an ICD-10 code for *device & instrument* (Y62.0–Y82.9) surgical misadventures. The data are not limited to an operation type. The control group is matched on patient demographics (Table 1).

**Table 1.** Patient and control demographics

	Control	D&I	WM
Age			
μ ± σ	47.28 ± 23.96	47.45 ± 23.91	50.44 ± 22.84
<18	897 (17.46%)	85 (15.83%)	991 (12.99%)
18–44	1083 (21.09%)	133 (24.77%)	1507 (19.76%)
45–64	1670 (32.52%)	158 (29.42%)	2658 (34.85%)
65+	1486 (28.93%)	161 (29.98%)	2472 (32.41%)
Race			
African American	1400 (27.26%)	155 (28.86%)	2483 (32.55%)
Caucasian	3471 (67.58%)	354 (65.92%)	4773 (62.57%)
Other	228 (4.44%)	23 (4.28%)	322 (4.22%)
Unknown	37 (0.72%)	5 (0.93%)	50 (0.66%)
Gender			
Female	2556 (49.77%)	302 (56.24%)	3834 (50.26%)
Male	2580 (50.23%)	235 (43.76%)	3794 (49.74%)
Ethnicity			
Hispanic	130 (2.53%)	16 (2.98%)	169 (2.22%)
Not Hispanic	4968 (96.73%)	519 (96.65%)	7417 (97.23%)
Unknown	38 (0.74%)	2 (0.37%)	42 (0.55%)

### Training pipeline

The CNN training pipeline is as follows:

1. Clean data with regular expressions and other string operations.
  - The code can be found in the “cleanTxt” function of the text_utils.py file.^15^ The operations include removal of special characters, conversion to ASCII, etc.
2. Stratify data by patient, except intentional overlap during the third use case to estimate information leakage.
3. Run 1-Cycle policy^15^^,^^16^
  - 10 iterations
  - In each iteration, split the data into 75% (train), 15% (validate), and 10% (holdout) sets.

### Use cases

We present 3 use cases:

1. Filter consistency
  - Does ranking by train vs validate set produce consistent results?
2. Performance improvement
  - Use RFBF and logit delta plots to identity filters that do not generalize.
  - set $FC_{k}=0$ for these filters.
3. Estimation of train-holdout information leakage via feature extraction.
  - Use RFBF to identify a filter that learns a feature which should not have cross-dataset instances. For this example: the 5-gram filter that extracts patient name, in the form of “patient name …”
  - For each sample, extract patient name from the ngram at the identified filter’s max pool location.
  - Calculate patient name overlap between train and validate.
  - Apply a–c. in simulations that increase overlap between train and holdout sets (0%, 10%, 30%, 50%).

## RESULTS

### Classification performance

The model achieved 0.78 ± 0.04 AUC (D&I), and 0.71 ± 0.01 AUC (WM) across 10 data splits.

### Use case 1: Filter consistency

The top 3 filters with the greatest mean logit difference on the *train set* for *WM* classification focus on patient names. The highest rank filters for the *holdout set* extracts history, post-operative diagnosis, revision, infection, and hardware (Figure 2). For *D&I*, different sort arrangements result in more consistent ranks with a focus on hardware removal, infection, and specimen distribution. However, when ranked by train set AUC, the first D&I filter extracts Electronic Health Record (EHR) template features (Supplementary Figure S1).

### Use case 2: Performance improvement

**Figure 3.**
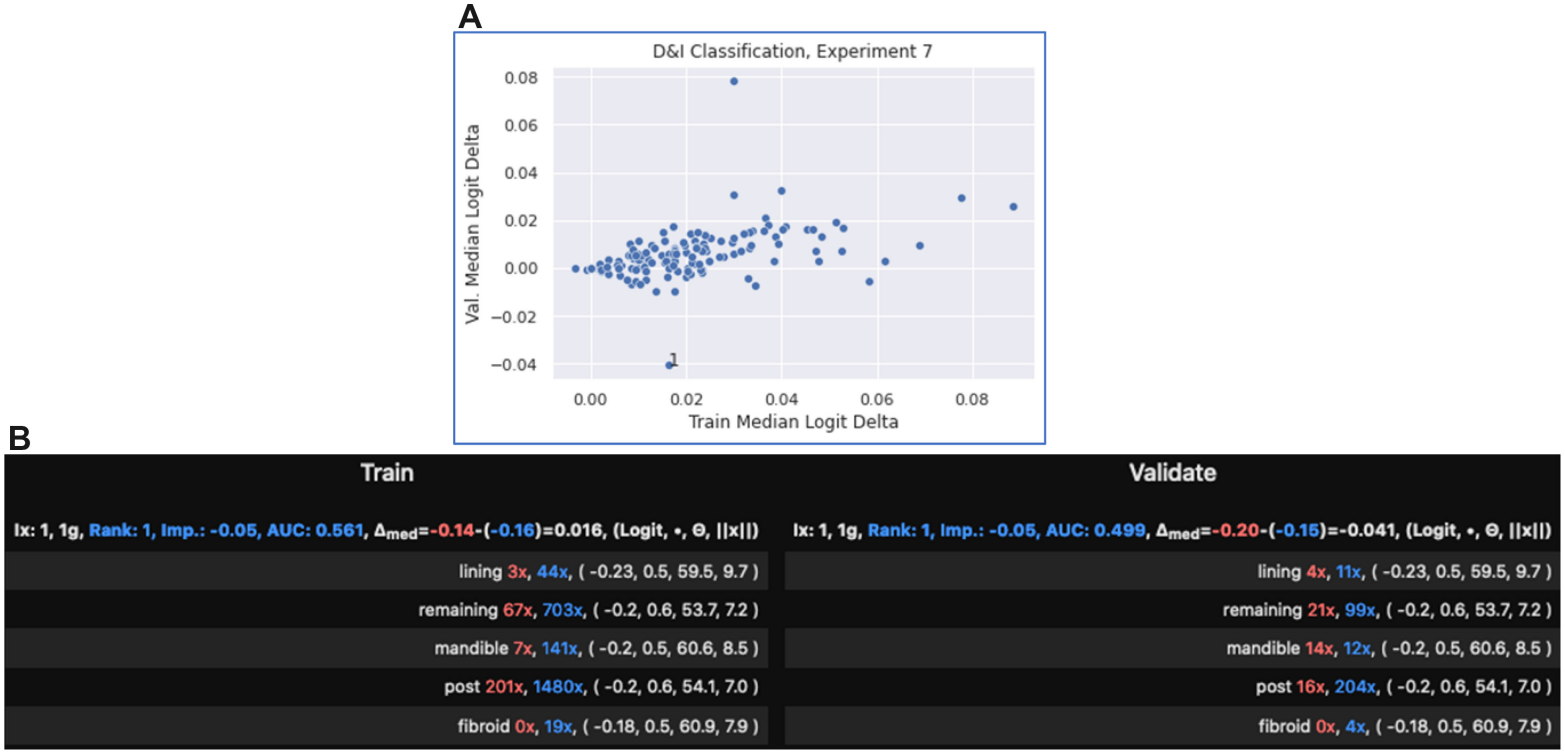
Experiment 7, D&I classification. (A) Top: Filter 1 is an outlier for validate set performance. (B) Bottom: Distribution drift between train (left) and validate (right).

A plot of the median logit deltas between the train and validate sets for experiment 7 (highest AUC, 0.839), D&I, reveals filter 1 as an outlier. Where “1” references the filter index in the ZF dictionary and classification layer. RFBF indicates distribution drift for the filter. For example, “mandible” is nearly control set exclusive in the train set, but it is slightly associated with patients in the validate set (Figure 3).

Removal of filter 1 led to an improvement of holdout AUC from 0.839 to 0.843.

### Use case 3: Estimation of Train-Holdout information leakage through feature extraction

The correlation between calculated and observed train/holdout overlap equaled 0.993 (D&I), and 0.999 (WM) (Supplementary Figure S2).

## DISCUSSION

### Classification performance

TextCNN surpasses chance on these challenging sets. Device & Instrument performance surpassed Without Mention. This may be because WM operative notes do not record the safety incident at time of operation.

### Use case 1: Filter consistency

RFBF indicates that many of the most activating features are overfit train features—template artifacts, patient names, etc. However, ranks by validate/holdout performance reveal relevant features.

Patient name-extraction filters rank highest on the train set for WM, hence X-AI techniques that rank by logit magnitude would emphasize patient name features. In comparison, a filter sort via discriminative performance on the validate set reveals informative features: revision, loose/painful hardware, and infection.

### Use case 2: Performance improvement

For D&I classification, the following 3-step process led to a holdout AUC improvement from 0.839 to 0.843. (1) Identify an outlier filter on a plot of validate-train logit deltas (Figure 3A); (2) confirm a train-validate distribution shift in RFBF; and (3) remove the filter. While the improvement is slight, it can impact model rank experiments. For example, this improvement is larger than the gap between second place TextCNN and first place 1-layer encoder in a recent research^8^ that compares neural network models for medical text classification.

A filter that overfits a concept may still provide a classification benefit if (1) it learns more than one concept; and (2) the proportions of the overfit feature do not favor one class over another on unseen data.

To prevent overfitting, the holdout set should only be analyzed at the end of experiment.

### Use case 3: Estimation of Train-Holdout information leakage through feature extraction

RFBF can serve as a starting point to identify features that inflate performance. For example, a data loader bug may load samples from the same patient in the train and validate/holdout sets. Such features are readily visible in RFBF because they tend to have high train set logit deltas. We present strong results for overfit estimation via patient-name extraction.

While patient names are ideally masked, the results show that names serve as informative data points to detect information leakage. We therefore propose a workflow that first uses RFBF to check for information leakage via patient name, and the researcher then continues analysis with preprocessed name masking if there is no evidence of leakage.

### Source code

The RFBF source code is on GitHub.^15^

The surgical datasets are not publicized due to Protected/Personal Health Information (PHI). Instead, the publicized sample is a set of 6726 PubMed abstracts (2268 positives, 4458 controls). The outcome variable is presence of a MESH term for Hepatitis. The model achieved an AUC of 0.966.

### Limitations and future research

Future research can improve upon this research.

- Include support for other DL libraries besides PyTorch.^3^
- Update RFBF to visualize other binary classification models that have a single, additive classification layer. For example, token contributions for each of the final classification layer neurons can be calculated via DeepLIFT.^17^ Given a final classification layer, each classification neuron still acts as a model that feeds an unweighted additive ensemble. And for each of these neurons (models), the extended RFBF could display both (1) the highest activating token patterns; and (2) the interclass discriminative capability. Unlike direct use of current X-AI techniques,^14^^,^^17^^,^^18^ this serves as a model diagnostic tool to verify each classification neuron via predictive performance and display of the learned features.
- Multi-class support: Red could correspond to a class under interest specified by the user, and blue corresponds to an aggregate of other classes. RFBF can display filter performance through metrics such as OvR (one vs rest) AUC.
- A modification of SHAP^14^ values to account for interclass differences, rather than subtract the overall mean from the activations.

### Summary

RFBF is a model interpretability toolkit that portrays (1) interclass discrimination performance for each of the neurons in the final classification layer, and (2) the grams that most activate the neurons. Discriminative capability is a more useful diagnostic for small and noisy datasets where trainset nuances can lead to large activations. We present 3 use cases for this diagnostic tool: filter consistency, performance improvement, and estimation of information leakage. Due to the prevalence of TextCNN for clinical text, this addresses an important research problem. Future work can extend RFBF to support models besides TextCNN (model interpretability); and interclass logit comparisons to assess discriminative performance may lead to a new approach to calculate feature values (model and sample interpretability).

## Supplementary Material

ooac112_Supplementary_Data

## Data Availability

The referenced surgical notes dataset is PHI-sensitive and not publicly available. The Red Flag/Blue Flag GitHub page contains a corpus of 6726 PubMed case reports (2268 positives with MESH term for Hepatitis C, 4458 controls). The dataset is composed of case reports of 5 MESH-term based queries pulled from the PubMed API: (1) “Cirrhosis,” (2) “Hepatitis C,” (3) “Hepatitis,” (4) “Non-alcoholic Fatty Liver Disease,” and (5) a set of queries with no specified MESH term. All queries subject to time constraints 2011 to 2018.
