# Supplementary material for: Red Flag/Blue Flag visualization of a common CNN for text classification
Source: JAMIA Open. 2023 Jan 16;6(1):ooac112. doi: 10.1093/jamiaopen/ooac112 (PMC9841396; doi:10.1093/jamiaopen/ooac112)
Supplement: ooac112_Supplementary_Data [file ooac112_supplementary_data.pdf]

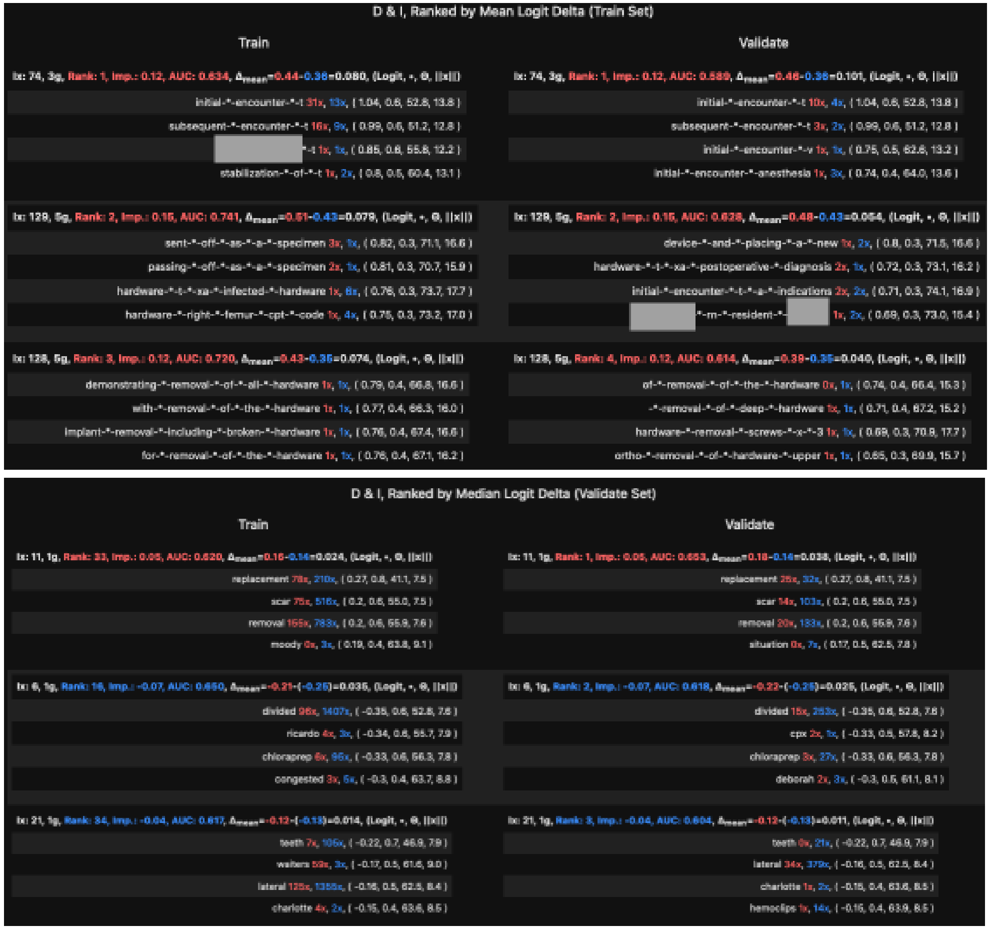

Supplemental Figure 1a: The top 3 filters ranked on Mean Logit Delta (train set) for classification of D&I.
Supplemental Figure 1b: Same as Figure 1a but ranked on Median Logit Delta (validate set). Note the consistent filters ranks across datasets and metrics compared to WM classification, which had lower performance.

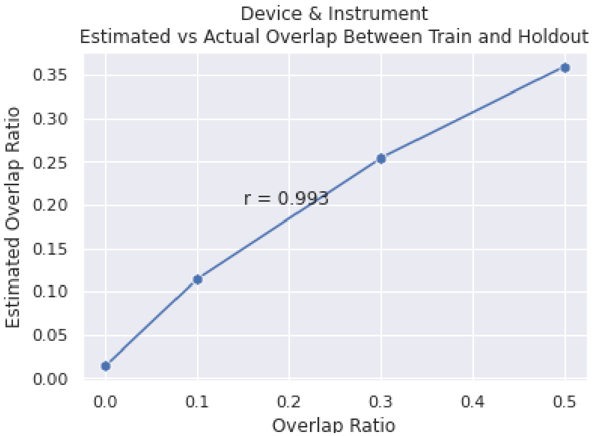

Supplemental Figure 2a: D&I (Device & Instrument); correlation between actual and estimated train/holdout overlap.

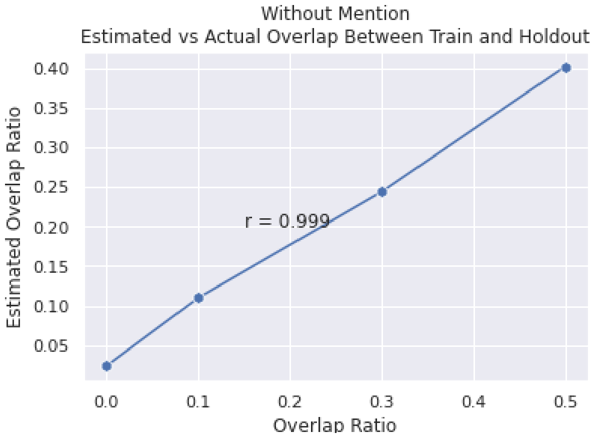

Supplemental Figure 2b: WM (Without Mention); correlation between actual and estimated train/holdout overlap.
